# Mammographic density and breast cancer: a comparison of related and unrelated controls in the Breast Cancer Family Registry

**DOI:** 10.1186/bcr3430

**Published:** 2013-05-25

**Authors:** Linda Linton, Lisa J Martin, Qing Li, Ella Huszti, Salomon Minkin, Esther M John, Johanna Rommens, Andrew D Paterson, Norman F Boyd

**Affiliations:** 1 Campbell Family Institute for Breast Cancer Research, Ontario Cancer Institute, 610 University Avenue, Toronto, ON M5G 2M9, Canada; 2 Cancer Prevention Institute of California, 2201 Walnut Avenue, Fremont, CA 94538, USA, and Division of Epidemiology, Department of Health Research and Policy, Stanford University School of Medicine,150 Governor's Lane, Stanford, CA 94305, USA, and Stanford Cancer Institute, 265 Campus Drive, Stanford, CA 94305, USA; 3 Program in Genetics and Genomic Biology, The Hospital for Sick Children, 555 University Avenue, Toronto, ON M5G 1X8, Canada; 4 Dalla Lana School of Public Health, University of Toronto, 155 College Street, Toronto, ON M5T 3M7, Canada

**Keywords:** Mammographic density, case-control study, overmatching, case control

## Abstract

**Introduction:**

Percent mammographic density (PMD) is a strong and highly heritable risk factor for breast cancer. Studies of the role of PMD in familial breast cancer may require controls, such as the sisters of cases, selected from the same 'risk set' as the cases. The use of sister controls would allow control for factors that have been shown to influence risk of breast cancer such as race/ethnicity, socioeconomic status and a family history of breast cancer, but may introduce 'overmatching' and attenuate case-control differences in PMD.

**Methods:**

To examine the potential effects of using sister controls rather than unrelated controls in a case-control study, we examined PMD in triplets, each comprised of a case with invasive breast cancer, an unaffected full sister control, and an unaffected unrelated control. Both controls were matched to cases on age at mammogram. Total breast area and dense area in the mammogram were measured in the unaffected breast of cases and a randomly selected breast in controls, and the non-dense area and PMD calculated from these measurements.

**Results:**

The mean difference in PMD between cases and controls, and the standard deviation (SD) of the difference, were slightly less for sister controls (4.2% (SD = 20.0)) than for unrelated controls (4.9% (SD = 25.7)). We found statistically significant correlations in PMD between cases (*n *= 228) and sister controls (*n *= 228) (r = 0.39 (95% CI: 0.28, 0.50; *P *<0.0001)), but not between cases and unrelated controls (*n *= 228) (r = 0.04 (95% CI: -0.09, 0.17; *P *= 0.51)). After adjusting for other risk factors, square root transformed PMD was associated with an increased risk of breast cancer when comparing cases to sister controls (adjusted odds ratio (inter-quintile odds ratio (IQOR) = 2.19, 95% CI = 1.20, 4.00) or to unrelated controls (adjusted IQOR = 2.62, 95% CI = 1.62, 4.25).

**Conclusions:**

The use of sister controls in case-control studies of PMD resulted in a modest attenuation of case-control differences and risk estimates, but showed a statistically significant association with risk and allowed control for race/ethnicity, socioeconomic status and family history.

## Introduction

Breast cancer is well known to cluster in families, which is thought to reflect inherited susceptibility [1]. However, despite extensive research only 20% to 25% of the excess risk of breast cancer in the first-degree relatives of women affected by the disease can be attributed to mutations in known genes [2]. Much remains to be learned about why having a family history of the disease is a risk factor for breast cancer. In previous work we have shown that variations in the radiological appearance of the breast, referred to as mammographic density, were associated with familial breast cancer [3].

Mammographic density varies among women of the same age and reflects differences in breast tissue composition. Stroma and epithelium attenuate X-rays more than fat and appear light on a mammogram, while fat appears dark [4]. The proportion of the mammogram occupied by radiologically dense tissue (percent mammographic density (PMD)) is strongly associated with an increased risk of breast cancer [5-7]. PMD varies with age, and among women of the same age according to height, weight [11,12], parity [13,14], menopausal status [15] and menopausal hormone use [16], (reviewed in [8,9] but these factors explain only 20% to 30% of the observed variance in PMD). Twin studies have shown that, after adjustment for these risk factors, additive genetic effects account for about 60% of the variance in PMD [1]. Women with a first-degree relative diagnosed with breast cancer have on average more extensive PMD than women of the same age with no family history. Furthermore, average PMD increases with the number of first-degree relatives diagnosed with breast cancer [10,11]. In previous work we found that PMD explained 14% (95% CI, 4 to 39%) of the association of family history (at least one affected first-degree relative) with breast cancer risk [3].

These findings suggest that PMD may play a role in familial breast cancer and that studies of PMD might provide insight into the etiology of familial breast cancer. In studies of familial breast cancer, the unaffected sisters of breast cancer cases might be used as controls and have the advantage of matching on complex variables such as family history, race/ethnicity and socioeconomic status [12-14], and perhaps age, which have all been associated with PMD and/or breast cancer risk. In addition, sisters would be expected to be better matched for unmeasured environmental risk factors than unrelated controls. However, the use of sister controls might also result in overmatching, and attenuate the case-control difference seen in studies using unrelated controls. The purpose of this study was to estimate the risks of breast cancer associated with PMD using controls related to cases and unrelated controls, and to compare the results obtained.

## Methods

### General method

We used mammographic images previously collected from three epidemiological studies investigating the heritability and genetics of PMD. From these studies we had available mammograms for at least two full sisters from a family, and an unrelated control. We formed triplets comprised of an invasive breast cancer case, a sister control, and an unrelated control, all matched according to age at the time of the mammogram. Only original mammographic films were used in the analysis and we selected one craniocaudal mammogram view from all participants, using the breast contralateral to the cancer of cases, and a randomly selected side for controls. Ethics approval was obtained from the University Health Network's Research Ethics Board and the Cancer Prevention Institute of California's Research Ethics Board.

### Study populations

Study participants were identified from three sources, including the Ontario and Northern California sites of the Breast Cancer Family Registry (BCFR) [15], the Weekend to End Breast Cancer in Toronto (a fund-raising walk), and the Canadian component of a twin study from which we randomly selected one twin [1].

The Breast Cancer Family Registry (BCFR) has been described in detail elsewhere [15]. The BCFR was established in 1995, with six participating sites from the USA, Canada, and Australia ascertaining families either from population-based cancer registries or from clinical settings. Population-based families were recruited by the Northern California Cancer Center; from the province of Ontario, Canada; and from the metropolitan areas of Melbourne and Sydney, Australia. Clinic-based families were recruited in the USA by Columbia University, New York, the Fox Chase Cancer Center, Philadelphia, and the Huntsman Cancer Institute at the University of Utah in Salt Lake City, Utah; and in Australia by the University of Melbourne and New South Wales Cancer Council in Melbourne and Sydney, Australia.

Population-based recruitment of incident cases of breast cancer reported to cancer registries in Northern California and Ontario were used in the present research. Both sites recruited cases and their family members selected according to age and the presence of a family history of breast and other cancers. All participants completed the same family history and epidemiology questionnaires and provided information on family history of breast and other cancers and epidemiologic risk factors for breast cancer, including height, weight, menopausal status and parity. All participants in addition completed a food frequency questionnaire, and provided a blood sample from which DNA was extracted. Informed consent was obtained from all participants for a larger study [16], which included the current analysis.

### Subject selection and data collection

#### Selection of cases

Cases were selected from the BCFR and were women with a personal history of unilateral invasive breast cancer, for whom a mammogram prior to the cancer diagnosis was available.

#### Selection of controls related to cases

Sisters selected from the BCFR were matched to cases on age at mammogram and selected if the mammograms were within five years of age and no later than up to one year from the epidemiological questionnaire administration. In situations where there was more than one unaffected sister in the family, the sister with the mammogram closest in age to that of the case was selected.

#### Selection of unrelated controls

The unrelated controls were selected from a previous twin study [1] and the Weekend to End Breast Cancer. They were also individually matched to cases based on age at mammogram screening and selected if the mammograms of the case and unrelated control were within five years of age.

A total of 228 triplets, including 686 individuals, was available for analysis. Of these, 179 case/sister pairs were from the Ontario BCFR, 49 case/sister pairs were from the Northern California BCFR, 11 unrelated controls were from the Weekend to End of Breast Cancer and 217 unrelated controls were from the twin study.

### Classification of menopausal status

Menopausal status was classified according to reported age at cessation of menstruation and the age at mammogram. If the age at mammogram was less than age at cessation of menstruation, or the age at mammogram was less than one year greater than the age at cessation of menstruation, the subject was classified as premenopausal. When the difference between age at mammogram and age when menstruation stopped was 12 months or more, the participant was classified as postmenopausal.

### Mammographic density measurement

All mammograms were digitized using a Lumisys model 85 digitizer (Lumisys, Sunnyvale, CA, USA) at a pixel size of 260 µm and 12 bits precision, and the digitized images were measured by one observer (NFB). The case-sister-control sets were always in the same read, and randomly distributed within that read. There was a total of eight reads of 110 images, also containing the within and between reliability images. Reliability was assessed both within and between reads using a 10% random selection of images and was 0.97 and 0.79 for within and between reads respectively. Measurement of mammograms has been described elsewhere [17]. Using the Cumulus 3 program (Canto, Berlin, Germany) an observer first marked the outer and inner edges of the breast, from which total breast area was then calculated. Using a thresholding tool, the observer outlined the dense area. The percentage of total area that is dense, or PMD, and the non-dense area were calculated.

### Statistical methods

The data analyzed consisted of three matched groups of 229 individuals, for a total of 687 participants. Data analysis was carried out using SAS (version 9.2 for Windows; SAS Institute Inc., Cary, NC, USA). The difference between PMD, dense area, non-dense area, and total breast area for the cases and both control groups were assessed using paired *t*-tests. We used Pearson correlation coefficient to examine the linear dependence of mammogram measurements on covariates between the cases and both control groups, separately. In addition, intraclass correlation coefficients (ICC) were calculated after adjustment for age, body mass index (BMI), menopausal status (pre or post), parity (parous and nonparous), hormone use (never, ever but not current, and current use) and case or control status.

We calculated inter-quintile odds ratios (IQOR) and 95% confidence intervals (CI) to assess the risk of breast cancer associated with square root transformed mammographic measures using conditional logistic regression, with each mammographic measure as a continuous variable. IQORs show the effect on breast cancer risk of change in transformed mammographic measures from the lowest to the highest quintile, adjusted for age, weight (kilograms), height (centimeters), parity, current menopausal hormone therapy (HT) use and menopausal status. All statistical tests were two-sided and the significance level was 0.05.

## Results

### Characteristics of subjects

Table 1 shows selected characteristics of the subjects. Cases, related controls and unrelated controls were similar for most of the characteristics examined. Notwithstanding age matching within five years, there were small but statistically significant differences in age at the time of mammogram between cases and both related (-0.68 months; *P *<0.0001) and unrelated (-0.21 months; *P *= 0.0003) controls. Sister controls were more similar to cases in height, weight and BMI than were unrelated controls. As a result of the method of selection of subjects from the BCFR, all sister controls had a family history of breast cancer, and a family history of breast cancer was reported by 48% of the cases and 23% of unrelated controls. A slightly greater proportion of sister controls were postmenopausal (45.2%) compared to cases and unrelated controls (both 44.7%). Ninety-two percent of the cases and their sisters, and 93% of unrelated controls were white Caucasian.

**Table 1:** Descriptive statistics for case and control subjects, and for case-control paired differences.

	Mean (SD)			Case - Sister control		Case - Unrelated control	
	
	Case N = 228	Control		Mean (SD) N = 228	*P *value^c^	Mean (SD) N = 228.	*P *value^c^
						
		Sister N = 228	Unrelated N = 228				
Age at mammogram (years)	50.7 (8.0)	51.4 (8.1)	50.9 (7.6)	- 0.68 (2.0)	<.0001	-0.21 (0.8)	0.0003
Weight (kg)	68.9 (13.7)	68.8^a ^(16.2)	67.0 (15.9)	0.15 (17.0)^a^	0.89	1.88 (20.1)	0.16
Height (cm)	163.3 (6.9)	163.4^b ^(6.2)	162.5 (6.8)	- 0.02 (6.8)^b^	0.96	0.81 (9.8)	0.22
Body mass index (kg/m^2^)	25.8 (4.7)	25.8^b^(5.8)	25.3 (5.4)	0.07 (6.0)^b^	0.87	0.48 (7.0)	0.30
Menopausal status (% post)	44.7	45.2	44.7	-0.5	0.89	0	1.00
Parous (% yes)	79.8	84.2	80.7	-4.4	0.19	-0.9	0.81
HRT ever used (% yes)	41.7	34.4^a^	37.3	7.3	0.08^c^	4.4	0.30
HRT current use (% yes)	29.8	22.4	29.8	7.4	0.04	0	1.00
Ethnicity (% white)	92.1	92.1	93.0	NA	NA	-0.9	0.72
Family history (% yes)	48	100	23	NA	NA	NA	NA

^a^N = 227; ^b^N = 226. For analysis, the overall averages were substituted for the missing values; ^c^paired *t*-test for continuous variables and McNemar's test for categorical variables. HRT, hormone replacement therapy; SD, standard deviation.

### Comparison of mammographic measures between cases and controls

**Figure 1:**
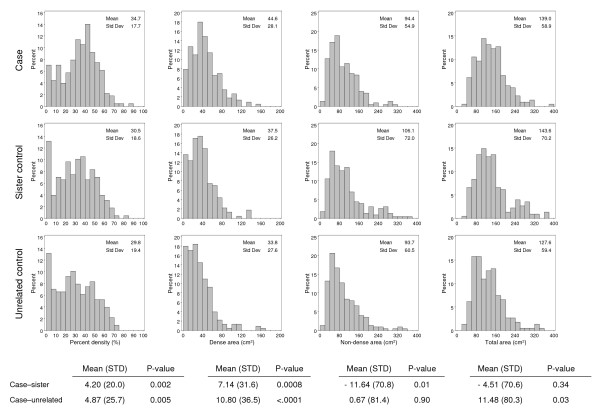
**Distribution of percent mammographic density, dense area, non-dense area and total area for cases, sister controls, and unrelated controls**. Shown are the means of the differences between cases and sister controls, and cases and unrelated controls. *P *is a *P *value from the paired *t*-test, two-sided.

Figure 1 shows the distributions of PMD, dense area, non-dense area and total breast area, in cases and the two control groups. Mean PMD was greatest in cases (34.7%), lower in sister controls (mean PMD = 30.5%) and least in unrelated controls (mean PMD = 29.8%). Cases also had more extensive dense tissue (mean 44.6 cm^2^) than sister (mean 37.5 cm^2^) and unrelated (33.8 cm^2^) controls. Non-dense area was 94.4 cm^2 ^in cases, 106.1 cm^2 ^in sister controls, and 93.7 cm^2 ^in unrelated controls. Total breast area was similar in cases (mean = 139.0 cm^2^) and sister controls (mean = 143.6 cm^2^) but was greater in cases than in unrelated controls (mean = 127.6 cm^2^).

The difference in mean PMD between cases and sister controls (mean = 4.20%; standard deviation (SD) = 20.0) was statistically significant (*P *= 0.002). The mean difference in PMD between cases and unrelated controls (mean = 4.9 %; SD = 25.7) was also significant (*P *= 0.005). The difference in dense area was also statistically significant between cases and sister controls (mean difference = 7.14 cm^2^; SD = 31.6; *P *= 0.0008) and between cases and unrelated controls (mean difference = 10.80 cm^2^; SD = 36.5; *P *<0.0001). The non-dense area was significantly different between cases and sister controls (mean = -11.64; SD = 70.8; *P *= 0.01), but not unrelated controls (mean = 0.67; SD = 81.4; *P *= 0.90). The difference in total breast area between cases and sister controls (mean = -4.51; SD = 70.6) was not statistically significant (*P *= 0.34), but was significantly different between cases and unrelated controls (mean = 11.48; SD = 80.3; *P *= 0.03).

### Correlation of mammographic density between cases, sister controls and unrelated controls

**Figure 2:**
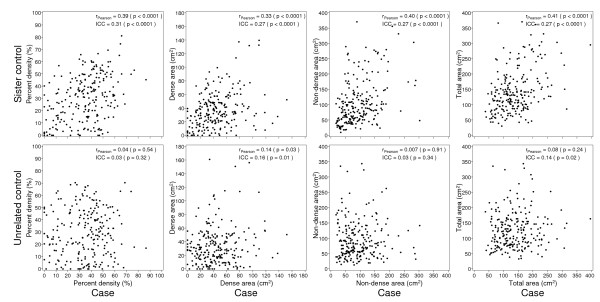
**Correlations of percent mammographic density, dense area, non-dense area and total area between cases and sister controls, and cases and unrelated controls**. *r *is a Pearson correlation coefficient.

As shown in Figure 2, the unadjusted Pearson correlation coefficient (r_pearson_) for related case-control pairs showed a moderate correlation in PMD (r = 0.39, *P *<0.0001), dense area (r = 0.33, *P *<0.0001), non-dense area (r = 0.40, *P *<0.0001), and total breast area (r = 0.41, *P *<0.0001). The ICCs, adjusted for the factors shown in the Footnote to Figure 2, were all statistically significant but were smaller than the corresponding Pearson correlations. The ICCs (adjusted for other risk factors) were 0.31 for PMD, and 0.27 for all the other comparisons.

For unrelated controls and cases, none of the correlations for PMD (r = 0.04, *P *= 0.54), non-dense area (r = 0.007, *P *= 0.91), and total breast area (r = 0.04, *P *= 0.28) were statistically significant. There was a weak positive and significant correlation for dense area (r = 0.14, *P *= 0.03). The ICCs were similar to the Pearson correlations, but weak statistically significant associations were seen for the dense area (ICC = 0.16, *P *= 0.01) and the total area (ICC = 0.14, *P *= 0.02), which may be chance findings.

**Figure 3:**
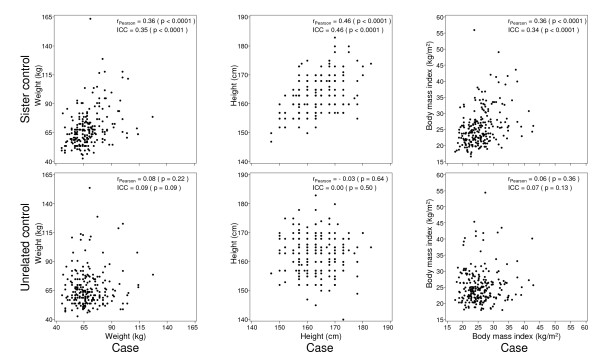
**Correlations of weight, height, and body mass index between cases and sister controls, and cases and unrelated controls**. *r *is a Pearson correlation coefficient.

Figure 3 shows correlations for measures of body size that are associated with variations in PMD between cases and sister controls and between cases and unrelated controls. Height was obtained by self-report and the distribution of values suggests that height was reported with rounding to whole numbers.

We found significant correlations between cases and sister controls (*P *<0.001) for weight, height and BMI, but these measures were not significantly correlated between cases and unrelated controls. The ICCs, adjusted for the factors shown in the Footnote to Figure 3, were all statistically significant, and similar to the Pearson correlations for cases and sister controls. ICCs comparing cases and unrelated controls were not statistically significant.

### Risk of breast cancer according to percent mammographic density and control group

Table 2 shows associations between square root transformed PMD and breast cancer risk, comparing cases to sister controls and unrelated controls, adjusting for the risk factor shown in the Footnote to the table. The coefficient for PMD (indicating the change in risk of breast cancer associated with change in one (square root transformed) unit in percent density) was 19% less for sister controls than for unrelated controls. The adjusted IQOR for PMD was correspondingly smaller for sister controls (2.19: 95% CI: 1.20, 4.00) than for unrelated controls (2.62; 95% CI: 1.62, 4.25) but both confidence intervals excluded unity. The analysis of the association of (square root transformed) dense area with risk showed that the coefficient was 37% smaller when sister controls were used but both control groups gave statistically significant associations of dense area with risk of breast cancer. Non-dense area was inversely and significantly associated with breast cancer when sister controls were used, but not with unrelated controls.

**Table 2:** Association of mammographic measures with risk of breast cancer when using sister controls or unrelated controls^a^

		Sister controls (*n *= 228 pairs)			Unrelated controls (*n *= 228 pairs)		
	Inter-quintile range^b^	Βeta (SE)	*P *value	Inter-quintile OR (95% CI)^c^	Βeta (SE)	*P *value	Inter-quintile OR (95% CI)
Percent density	10.0 - 48.1	0.2062 (0.0810)	0.01	2.19 (1.20, 4.00)	0.2538 (0.0648)	<.0001	2.62 (1.62, 4.25)
Dense area	11.0 - 50.6	0.1499 (0.0607)	0.01	1.77 (1.12, 2.78)	0.2365 (0.0510)	<.0001	2.46 (1.68, 3.59)
Non-dense area	45.1 - 136.7	-0.1364 (0.0598)	0.02	0.51 (0.28, 0.91)	-0.0142 (0.0520)	0.78	0.93 (0.56, 1.55)
Total area	77.3 - 165.0	-0.0591 (0.0633)	0.35	0.79 (0.47, 1.31)	0.1483 (0.0596)	0.01	1.84 (1.14, 2.97)

^a^All coefficients are based on square root transformed mammogram measures; ^b^inter-quintile range from unrelated controls, on the untransformed mammographic measures; ^c^inter-quintile odds ratio (95% confidence interval (CI)) is based on the inter-quintile range of square root transformed mammogram measures, adjusted for age at mammogram, parity, menopausal status, hormone usage (never, ever but not now, current using), and body mass index. OR, odds ratio; SD, standard error.

Mammograms in sisters were almost all carried out after the corresponding mammogram in the case. Restriction of the analyses to those case-sister pairs where the mammogram of the sister control was carried out after that of the case did not change the results (data not shown) and provides no support for the hypothesis that mammographic density in the sister control was influenced by changes in lifestyle prompted by the diagnosis of breast cancer in the case.

## Discussion

We carried out this study to determine if the risk of breast cancer associated with PMD in a case-control study would differ when controls were related or unrelated to the cases. Specifically, we sought to determine whether the selection of controls related to cases would introduce 'overmatching' and attenuate estimates of breast cancer risk associated with PMD. Our results showed expected correlations between cases and sister controls for PMD and the dense and non-dense areas of the mammogram, as well as for height, weight and BMI, all variables that are associated with PMD. As expected, cases and unrelated controls in general showed no significant correlations for these variables.

We have found that percent density is associated with breast cancer risk whether unrelated or sister controls were used, with modest attenuation of effect when sister controls were used. We think there are likely two principal reasons for these findings. First, PMD is a very strong risk factor for breast cancer [5], and as shown here, the mean difference in PMD between cases and sister controls and unrelated controls was only slightly smaller for sister controls than for unrelated controls (4.2% and 4.9% respectively). Further, the adjusted correlation in PMD between cases and sisters was modest (ICC = 0.31) and similar to the adjusted correlation in PMD (0.28) previously seen between dizygous twin sisters [1]. In the absence of both of these features, it is likely that there would be greater attenuation of the association of PMD with risk of breast cancer. Our results suggest that these factors should be considered before using related controls in studies of other phenotypes.

PMD is influenced by several factors that are also associated with breast cancer risk, including age, menopausal status, race/ethnicity, and a family history of breast cancer. When designing a study, controlling for some of these factors such as age and parity is straightforward, while others, such as family history and socioeconomic status, are more difficult. There are few suitable options for the selection of a control group, and the two that were investigated here were the use of age-matched, unrelated paired controls and a control group consisting of age-matched sisters of breast cancer cases.

Our results suggest that sisters are suitable controls for case-control studies examining factors associated with mammographic density. Coefficients and odds ratios for the association of PMD with breast cancer showed evidence of modest attenuation when sister controls were used, controlling for all covariates, although the confidence intervals of the odds ratios overlapped substantially with those for unrelated controls. As expected, correlations for height and weight were larger between cases and sister controls than for unrelated control. BMI is known to be a negative confounder of the effect of PMD with risk of breast cancer [18], and in these data adjustment for BMI had a larger effect than other covariates on the risk estimates for unrelated controls (data not shown).

## Conclusions

The use of sister controls in case-control studies of PMD resulted in a modest attenuation of case-control differences and risk estimates, but showed a statistically significant association with risk. These results are the consequence of the strong association of the mammographic density phenotype with breast cancer and the modest correlation in PMD between sisters. The use of sister controls for studies of mammographic density has the advantage of controlling for race/ethnicity and family history of cancer with little compromise in the case-control differences seen in PMD.

## Abbreviations

BCFR: Breast Cancer Family Registry; BMI: body mass index; CI: confidence interval; HRT: hormone replacement therapy; ICC: intraclass correlation coefficient; IQOR: inter-quintile odds ratio; PMD: percent mammographic density; SD: standard deviation; SE: standard error.

## Competing interests

The authors declare that they have no competing interests.

## Authors' contributions

LL carried out the design of the study, assisted in the analysis of the data and drafted the manuscript.

LM assisted in the interpretation of the results and the revision of the manuscript. QL, EH and SM performed the statistical analysis and assisted with data interpretation. EJ and JR participated in the acquisition of data and the revision of the manuscript. AP participated in the interpretation of the results and the revision of the manuscript. NB conceived of the study design, made mammographic measurements, and participated in the interpretation of the results. All authors participated in the drafting and revision of the manuscript. All authors read and approved the final manuscript.

## Authors' information

ADP holds a Canada Research Chair in the Genetics of Complex Diseases.
